# Buildings Contemplated and in Progress

**Published:** 1914-01-03

**Authors:** 


### Buildings Contemplated.

Barnet.—The Guardians have issued instructions to Messrs. Williams and Fox to submit plans for a new infirmary to the Local Government Board for approval. The plans provide for 192 beds at a cost of £80 per bed. The Guardians have resolved to build a separate Home for children under three years of age.

Bath.—The Corporation Sanitary Committee have resolved to obtain tenders for an addition to the Statutory Hospital. The estimated cost is £1,000, and the plans are by Mr. Gardiner.

Buckinghamshire.—The County Council has resolved to convene a conference of representatives of Amersham and Eton Rural District Councils, and Beaconsfield, Chesham, and Slough Urban District Councils, to consider the practicability of pooling the existing hospital accommodation and sites, with a view to formulating an amalgamation scheme for the treatment of (*a*) smallpox, and (*b*) other infectious diseases on separate sites.

Coalville.—The Urban District Council has approved plans for a tuberculosis dispensary for the Leicestershire County Council.

Finchley.—The Local Government Board has approved Chester Villa as a dispensary for the treatment of tuberculosis.

Hawkmoor.—The Devon County Council has agreed to erect new buildings for the county sanatorium at Hawkmoor. The estimated cost is £18,000, towards which the Local Government Board will make a grant of three-fifths.

Hemel Hempstead.—The Isolation Hospital Board has decided to apply to the Local Government Board for sanction to borrow £6,000 for a new hospital.

